# Single‐cell transcriptome and chromatin accessibility mapping of upper lip and primary palate fusion

**DOI:** 10.1111/jcmm.70128

**Published:** 2024-10-11

**Authors:** Sini Cai, Ningbei Yin

**Affiliations:** ^1^ The Department of Cleft Lip and Palate of Plastic Surgery Hospital Chinese Academy of Medical Sciences and Peking Union Medical College Beijing China; ^2^ Medical Cosmetic Center of Dermatology Hospital of Southern Medical University Guangdong Provincial Dermatology Hospital Guangzhou China

**Keywords:** development, primary palate, single cell RNA sequencing, single‐cell ATAC sequencing, upper lip

## Abstract

Cleft lip and/or primary palate (CL/P) represent a prevalent congenital malformation, the aetiology of which is highly intricate. Although it is generally accepted that the condition arises from failed fusion between the upper lip and primary palate, the precise mechanism underlying this fusion process remains enigmatic. In this study, we utilized transposase‐accessible chromatin sequencing (scATAC‐seq) and single‐cell RNA sequencing (scRNA‐seq) to interrogate lambdoidal junction tissue derived from C57BL/6J mouse embryos at critical stages of embryogenesis (10.5, 11.5 and 12.5 embryonic days). We successfully identified distinct subgroups of mesenchymal and ectodermal cells involved in the fusion process and characterized their unique transcriptional profiles. Furthermore, we conducted cell differentiation trajectory analysis, revealing a dynamic repertoire of genes that are sequentially activated or repressed during pseudotime, facilitating the transition of relevant cell types. Additionally, we employed scATAC data to identify key genes associated with the fusion process and demonstrated differential chromatin accessibility across major cell types. Finally, we constructed a dynamic intercellular communication network and predicted upstream transcriptional regulators of critical genes involved in important signalling pathways. Our findings provide a valuable resource for future studies on upper lip and primary palate development, as well as congenital defects.

## 1 INTRODUCTION

The maxillary process (MxP), lateral nasal process (LNP) and medial nasal process (MNP) in mice converge at a trifurcated seam designated as the lambdoidal junction.^1^, ^2^ This junction is highly susceptible to abnormalities during embryonic development, and a defective fusion at this seam gives rise to cleft lip and palate (CL/P), among the most prevalent congenital disorders.^3^ During embryogenesis, the tissue at the lambdoidal junction orderly transforms into the upper lip and primary palate. Given the substantial similarity in the developmental process between humans and mice,^4^, ^5^ the latter serves as an ideal model for a comprehensive characterization of cellular and molecular alterations throughout the fusion process.

In mice, the fusion of LNPs and MNP occurs at the oral aspect of the nasal fossae on embryonic day 10.5 (E10.5), accompanied by the fusion of the MxP with the LNP and MNP at the base of the nasal groove. Consequently, the tissue at the lambdoidal junction is a composite of cells originating from the MxP, MNP and LNP. The fusion between the upper lip and primary palate is completed by embryonic day 12.5 (E12.5). Various genes, including Tp63, Msx1, Irf6 and Fgfr1, among others, and developmental signalling pathways such as Wnt, notch, Shh, FGF and BMP, among others, are upregulated during this process, coordinating the fusion process in the correct sequence.^6^, ^7^ However, previous studies, conducted using bulk tissues, were unable to precisely identify the crucial molecules or signals that facilitate the development, differentiation and other biological functions of distinct cell types. In this regard, a recent investigation delved into the spatial distribution of subgroups within mesenchymal and ectodermal cells in E11.5 mouse lambdoidal junction tissues. This study characterized cell populations both within and surrounding the fusion zone,^8^ thereby laying the ground work for our subsequent exploration of cell type heterogeneity, cell‐specific transcriptional factors, cellular transitions and cell‐to‐cell communication throughout the entire fusion process. This exploration will involve single‐cell sequencing of mouse lambdoidal junction tissues at multiple time points. It is widely acknowledged that epigenetic regulation plays a pivotal role during development,^9^ yet the epigenetic modifications underlying the fusion of the upper lip and primary palate remain enigmatic. scATAC‐seq, a powerful tool, enables the profiling of single‐cell chromatin accessibility,^10^ potentially aiding in the prediction of transcriptional regulators of downstream target genes. In the present study, we utilized scRNA‐seq and scATAC‐seq on lambdoidal junction tissues derived from mouse embryos at the E10.5 (fusion commencement), E11.5(fusion) and E12.5 (fusion completion) stages. This approach aimed to identify the transcriptional and chromatin accessibility profiles of primary cells involved in upper lip and primary palate development. In addition, we conducted transcriptional and chromatin accessibility dataset profiling to demonstrate heterogeneous alterations in major cell types, dynamic cell‐to‐cell communication and cell‐type‐specific chromatin accessibility changes throughout the fusion process of the upper lip and primary palate.

## 2 METHODS

### 2.1 Ethics

All animal operations were approved by the Animal Ethics Committee of the Plastic and Surgery Hospital, Peking Union University, China (approval number: EAEC 2021‐012).

### 2.2 Samples

Lambdoidal junction tissues were obtained from day 10.5, 11.5 and 12.5 embryos of C57BL/6J mice, as previously described (Figure S1).^11^ As the clarity of the lambdoidal junction is lost at E12.5, scanning electron microscopy images of the nostril were used as a reference during microdissection.^12^ As the lambdoidal junction tissues provided few cells, 20–22 embryos were pooled and 3 replicates (20–22 embryos pooled per replicate) were used at each of the 3 time points. Tissue microdissection and single cell preparation were performed as described previously.^13^ For each sample, single cells (obtained by tissue digestion) were encapsulated by oil droplets for scRNA‐seq; the other parts of single cells were subjected to nuclear extraction for scATAC‐seq.

### 2.3 Single‐cell RNA and ATAC sequencing library preparation

For scRNA‐seq, the single cell suspension was loaded into Chromium microfluidic chips with 3′ v3 chemistry and barcoded using a Chromium controller (10× Genomics). RNA from the barcoded cells was then reverse‐transcribed and sequencing libraries were constructed using the Chromium Single Cell 3′ v3 reagent kitand sequencing was then performed using the Illumina platform. For scATAC‐seq, the single cell nucleus suspension was loaded into Chromium microfluidic chips for ATAC sequencing and barcoded using the Chromium Controller (10X Genomics). ScATAC‐Seq library was prepared following the 10X Genomics single‐cell ATAC‐Seq solution using protocol supplied by the manufacturer. Sequencing was then performed using the Illumina platform. All kits were used according to the manufacturer's instructions.

### 2.4 Quality control, dimension‐reduction and clustering for scRNA‐seq data

Raw reads were processed using Cell Ranger v6.1.0 software to generate gene expression profiles. Quality control analysis was then performed using the Scanpy package (v1.9.2),^14^ and dimensionality reduction and clustering were performed using Python 3.7. The expression matrix for each sample dataset was filtered by discarding the following: (1) cells containing <200 or the top 2% gene counts, (2) cells containing the top 2% unique molecular identifier (UMI) counts, (3) cells having a mitochondrial content of >20% and (4) genes expressed by less than 5 cells. After filtering, 76,738 cells were retained for downstream analyses, with an average of 3293 genes and 12,474 UMIs per cell. The top 2000 variable genes were selected for principal component analysis, and the top 20 principal components were retained; 8 cell clusters were determined using the Louvain algorithm with a resolution of 1.2. The data were further visualized using Uniform Manifold Approximation and Projection (UMAP). Potential batch effects among samples were corrected using Harmony v1.0^15^; the FindMarkers() function was then used to identify marker genes of different clusters. The cells from a specific cluster were extracted and re‐clustered with a resolution of 0.8, each, to obtain a high‐resolution map of mesenchymal and ectodermal cells. Any clusters enriched with multiple cell type‐specific markers were identified as cell doublets and removed for downstream analysis.

### 2.5 Differentially expressed genes (DEGs) and pathway enrichment analysis

Default settings of the scanpy.tl.rank_genes_groups() function were used to identify differentially expressed genes (DEGs) based on Wilcoxon rank sum test results. Genes expressed in more than 10% cells and with |avg_logFC| values of >1 were considered to be differentially expressed; *p*_val_adj values of <0.05 were considered significant. Gene Ontology (GO) and Kyoto Encyclopedia of Genes and Genomes (KEGG) analyses were performed using the clusterProfiler R package to evaluate the biological functions of cell‐type specific DEGs^16^; *p*_val_adj values of <0.05 were considered significant.

### 2.6 Pseudotime trajectory analysis

The Monocle2 (v2.12.0) algorithm was used to analyse the pseudotime of single cells and order cells along a trajectory.^17^ The counts matrix was used as the input, and the cells were annotated with cluster numbers identified using the Seurat package. The size factors and dispersions of our data were then estimated and pseudotime‐dependent genes were identified using the differentialGeneTest function of Monocle2 (qval <0.1). Pseudotime‐dependent genes were divided based on different expression patterns and GO analysis was performed for each pattern using the ToppGene tool (*p*‐value <0.05).^18^ The top 2000 highly variable genes were then selected and dimensionality reduction was performed using the DDRTree approach. The plot_cell_trajectory() function was subsequently used for visualizing the trajectory.

### 2.7 Trajectory switch gene analysis

Genes that switched on and off along the pseudotime were analysed using the GeneSwitches (V0.1.0) tool^19^; this helped determine the order of gene expression and function during cell state transitions. Logistic regression was used to model genes with a distinct bimodal ‘on–off’ distribution; the time point at which the fitted line crossed the probability threshold of 0.5 was estimated to be the switching point. The top switch genes with high McFadden's Pseudo R^2 values were plotted along the pseudo‐timeline using the plot_timeline_ggplot() function. Pathway analysis was then performed using the find_switch_pathway() function to better understand the function of switch genes; gene sets from the GO, KEGG and Molecular Signatures Database hallmark pathways were included. The reduce_pathways() function was used with the rate fixed at 0.8 to remove redundant pathways, and the plot_pathway_density() function was used to plot the top significantly altered pathways and order them based on the switching time.

### 2.8 Transcription factor (TF) regulatory network analysis

Transcription factor regulatory network analysis was performed with pySCENIC (v0.11.0),^20^ using scRNA data and transcription factors from the Animal Transcription Factors Database. The genes among specific clusters with the data matrix were used as input. Based on the matrix, the GRNBoost2 algorithm was used to construct co‐expression modules between transcription factors and potential target genes (filtered by default parameters). In each module, the potential target genes with significant motif enrichment for the corresponding TF were regarded as direct target genes. The TF and all its direct target genes were defined as a regulon. Cell clusters were identified and clustered again using the Seurat R package; the active matrix of regulons was used as input. Cells of each regulon were determined to be active according to the threshold determined by the distribution. Regulon activity quantification was performed using the AUCell R package and regulon specificity scores were calculated to identify the cluster‐specific regulons.

### 2.9 Cell–cell interaction analysis

Intercellular communication networks were constructed with CellChat (version 0.0.2) using scRNA‐seq data.^21^ Cell information matching was performed after creating a CellChat object, and the matching receptor inference calculation was performed after setting the ligand‐receptor interaction database.

### 2.10 scATAC‐seq data processing and clustering

Raw data were converted to the FASTQ format using the command, ‘cellranger‐atac mkfastq’ (10x Genomics, v.2.1.0). The scATAC‐seq reads were aligned to the GRCm38 (mm10) reference genome and quantified using the command, ‘cellranger‐atac count’ (10x Genomics, v.2.1.0). Cells with less than 1000 sequencing fragments or transcription start site enrichment scores of <4 were excluded, and bin regions overlapping with ENCODE blacklisted regions were removed. The iterative latent semantic indexing approach was then used to reduce the dimensionality of the sparse insertion counts matrix, and no batched was carried out to preserve wak biological features. Canonical correlation analysis was then performed to match scRNA and scATAC data and clustering was performed using the addClusters() function; the data were visualized using UMAP.

### 2.11 Integration of scRNA and scATAC data

The integration framework of Seurat (v4) was used to identify pairs of corresponding cells between data obtained by the two modalities; this helped interpret the scATAC‐seq data. The running environments for the packages used are the R language environments containing the seurat and ArchR packages. The scRNA‐seq dataset was used as the reference to train the classifier and each of the scATAC cells were assigned to types determined by scRNA. That is, shared correlation patterns between scATAC‐seq gene activity and scRNA‐seq gene expression were identified using the FindTransferAnchors function of Seurat (reduction = ‘pcaproject’). The cell type label of each cell from the scATAC‐seq data was then predicted using the TransferData function (weight. reduction = ‘lsi’ and dim 1:20). Finally, a total of 85,896 cells were retained after filtering based on transcription start site enrichment scores of >4 and fragment numbers of >1000. The filtered scATAC‐seq object was reprocessed using latent semantic indexing and clustered using the smart local moving algorithm. The Jaccard index was used to assess the consistency between cell identities, predicted by label transfer and curated annotations based on gene activities of known markers.

### 2.12 Peak calling

Peak calling was performed using MACS2 software, based on aggregated insertion sites from all cells. A consensus set of non‐overlapping peaks was acquired by retaining the peak with the highest score from each set of overlapping peaks. Briefly, the peaks were ranked by their significance and the most significant peak was selected; any peaks that directly overlapped with the most significant peak were excluded. This process was repeated until no more peaks existed.

### 2.13 Analysis of scATAC gene scores/ transcription factor activity

Gene expression (also known as the gene score) and TF motif activity were estimated from scATAC data using the ArchR package, and the addGeneScoreMatrix() function was used to calculate gene scores. The addMotifAnnotations() function was used to conform motif presence in the peak set of the JASPAR2022 motif dataset. The addDeviationsMatrix() function was then executed to calculate TF activity across all motif annotations (based on chromVAR).

### 2.14 Immunostaining

Immunostaining was performed as described previously.^22^ After antigen retrieval and blocking with serum for 1 h, the slices were incubated overnight with the primary antibodies at 4°C; these included: anti‐Epcam (Cell Signaling), anti‐Col1a2 (Santa Cruz), anti‐Hbb‐y (Immunoway) and anti‐Emcn (Immunoway), anti‐Tfap2b (Santa cruz), anti‐Dlk5 (Abcam), anti‐Igfbp5 (Santa cruz). The slices were then incubated with related secondary antibodies for 1 h at room temperature after washing thrice with phosphate buffered saline (PBS). Following incubation, they were washed thrice with PBS (5 min at each instance). Autofluorescence was removed using the Vector® True VIEW™ Autofluorescence Quenching Kit (Vectorlabs; SP‐8400), and the slides were washed with PBS for 5 min; they were then incubated with diamidino‐phenyl‐indole for 30 min. Images were acquired using laser scanning confocal fluorescence microscopy.

### 2.15 Differential analysis

The peak intensity was characterized by log2 values of the normalized read counts, and the *t*‐ and Benjamini‐Hochberg multiple tests were used to evaluate the *p* value and false discovery rate. *p* values of <0.05 were considered statistically significant.

## 3 RESULTS

### 3.1 Heterogeneity alterations of mesenchymal cells throughout the process of upper lip and primary palate fusion

**FIGURE 1:**
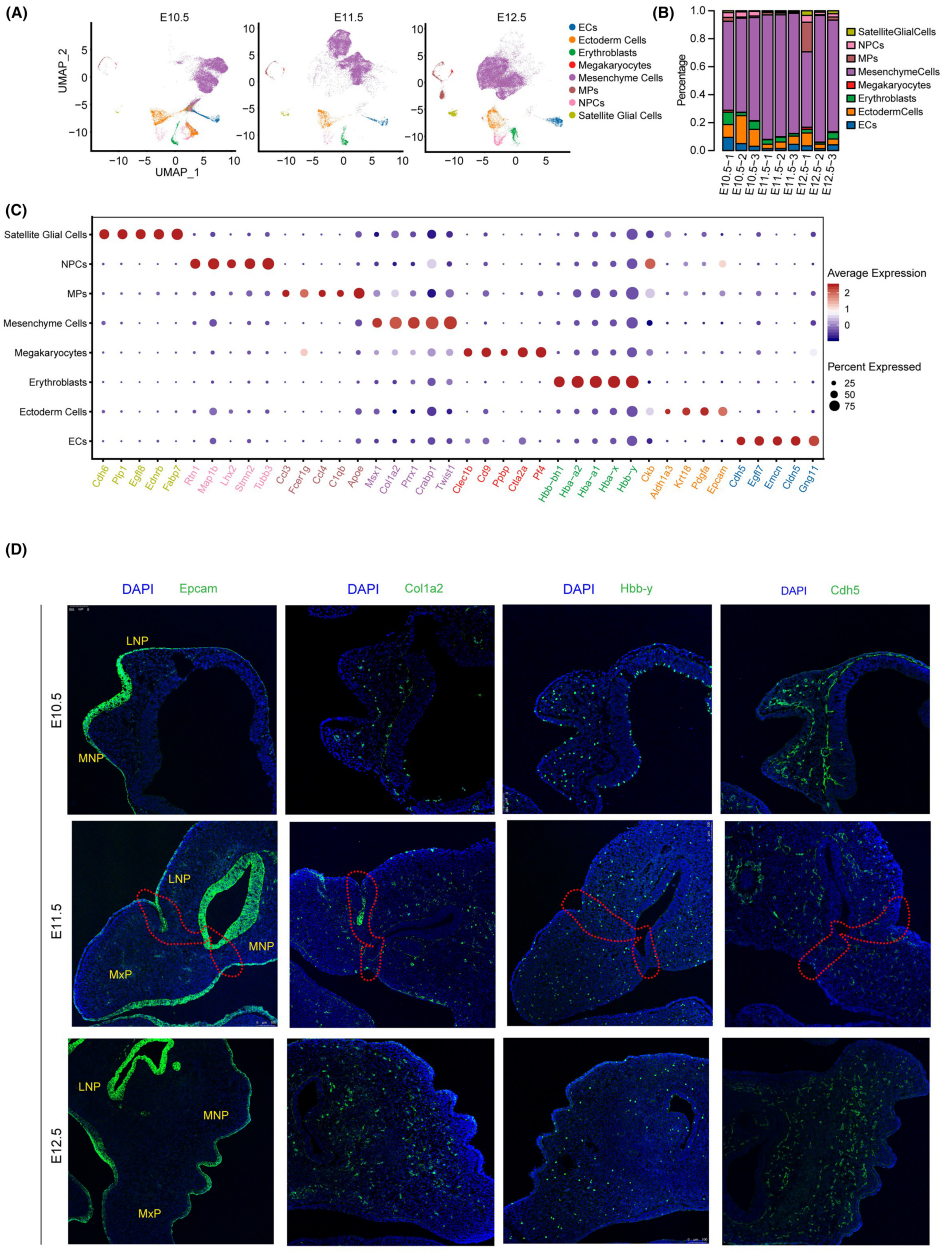
scRNA‐seq atlas of cell groups during upper lip and primary palate development. (A) Uniform manifold approximation and projection (UMAP) plot for lambdoidal junction tissue cells, derived from E10.5 (*n* = 3), E11.5 (*n* = 3) and E12.5 (*n* = 3) mice. (B) Comparison of the proportion of cells in E10.5 (*n* = 3; labelled as E10.5–1, E10.5–2, E10.5–3), E11.5 (*n* = 3) and E12.5 (*n* = 3) lambdoidal junction tissues. (C) Dot plot showing the expression of top five marker genes for each cell type. The node size related to the positive rates, and the colour key represents the expression levels of maker genes. (D) Immunofluorescence assay of representative marker genes for major cell types. Gene, green; nucleus, blue. LNP, the lateral nasal; MNP, the medial nasal; MxP, the maxillary prominence. The fusion junction was marked with a yellow dashed line. ECs, endothelial cells; NPCs, nuclear progenitor cells; MPs, mononuclear phagocytes.

A comprehensive evaluation of cellular and gene expression alterations during the development of the upper lip and primary palate was conducted using scRNA analysis. Tissue samples were obtained from the lambdoidal junction (formed by the MxP, LNP and MNP) of mouse embryos at E10.5 (*n* = 3), E11.5 (*n* = 3) and E12.5 (*n* = 3) stages (Figure S1). A total of 76,738 cells passed quality control analysis, exhibiting an average of ~3293 genes per cell and ~12,474 UMIs per cell. To eliminate batch effects among samples, the Harmony method was employed. UMAP analysis of the batch‐corrected datasets revealed eight distinct cell clusters (Figure 1A). Based on DEGs (Figure 1C, Table S1) and published canonical marker genes (Table S2), these clusters were manually annotated as mesenchymal, ectodermal and neural progenitor cells. Notably, mesenchymal cells were the predominant cell type in the lambdoidal junction tissues between E10.5 and E12.5 (Figure 1B,D).

**FIGURE 2:**
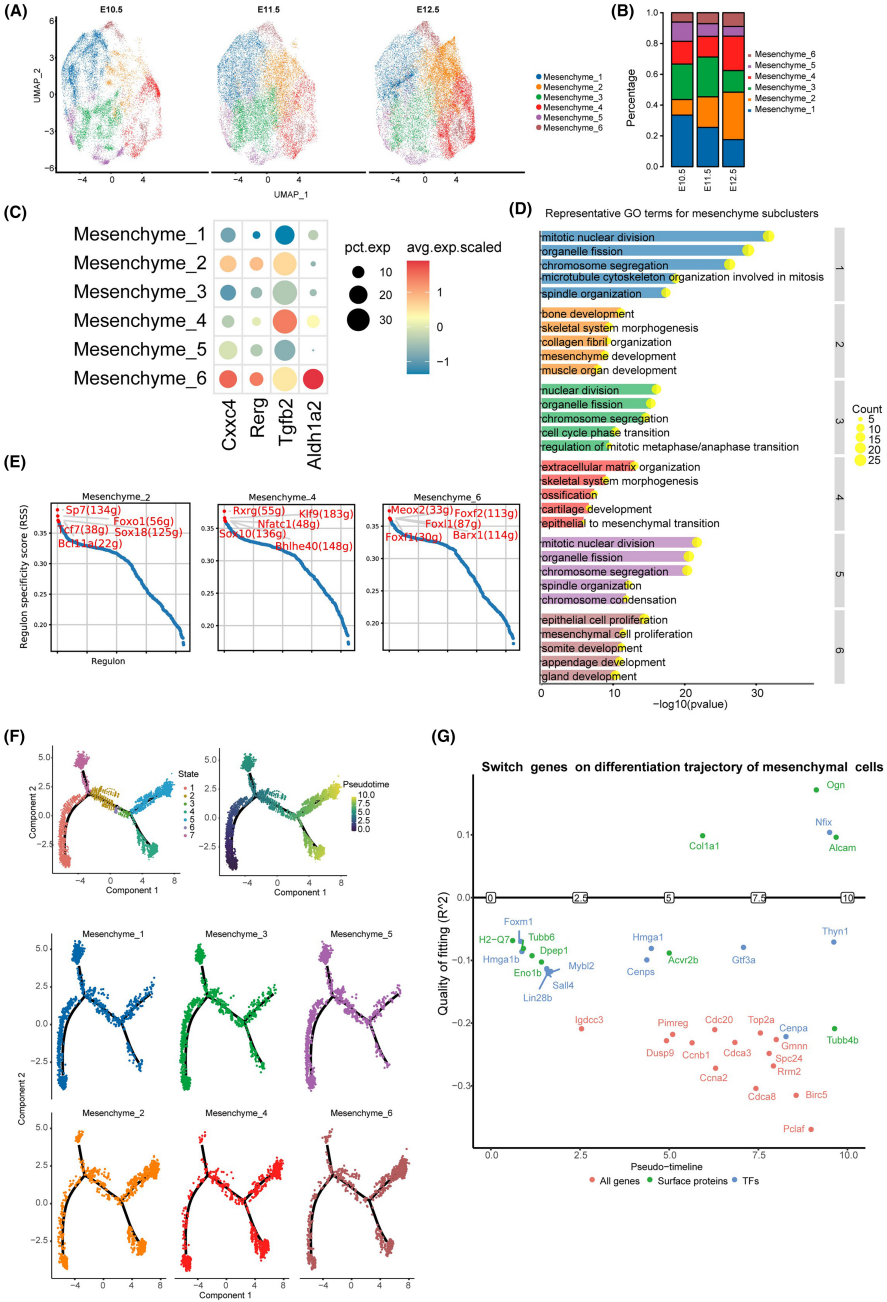
Heterogeneity changes of mesenchymal cells. (A) UMAP plots displaying six subsets of mesenchymal cells. (B) Bar diagrams presenting the ratio of subsets of mesenchymal cells. (C) Dot plot showing the expression of selected marker genes for subgroups of mesenchymal cells. Circle size represents percentage of cells expressing the given marker gene; Red colour represents high expression while blue colour represents low expression. (D) The gene ontology analysis of subsets of mesenchymal cells. (E) Regulon specificity score for mesenchyme subset 2, 4 and 6, and the top five highest scoring regulons are highlighted in red. pySCENIC provides standard functionality to calculate the scores. (F) Mesenchyme cells were projected onto the different cell states (upper, left), and the pseudotime icons was shown (upper, right). Cellular differentiation trajectory for each subset of mesenchymal cells were shown in the lower. (G) Plot of switch genes on the differentiation trajectory of mesenchymal cells. Genes above the horizontal line were switched‐on, while genes below the line were switched‐off.

The fusion of the upper lip and primary palate involves epithelial‐to‐mesenchymal transition and mesenchymal proliferation. Upon completion of the fusion process, mesenchymal cells differentiate into skeletal, muscle and connective tissue cells. In our study, mesenchymal cells were further classified into six subgroups. The proportions of sub‐mesenchyme 2 (Cxxc4^+^, Rerg^+^), sub‐mesenchyme 4 (Tgfb2^+^) and sub‐mesenchyme 6 (Cxxc4^+^, Rerg^+^ and Aldh1a2^+^) demonstrated a rise from E10.5 to E12.5 (Figure 2A–C; Figure S2A; Tables S3 and S4). The differentially expressed genes (DEGs) within mesenchymal subsets 2 and 4 were primarily implicated in skeletal system morphogenesis and epithelial to mesenchymal transition, respectively. Conversely, those in mesenchymal subset 6 were primarily associated with epithelial and mesenchymal cell proliferation (Figure 2D). Transcriptional factor regulatory network analysis revealed that mesenchymal subset 2 specifically expressed *SP7*, a bone marker gene (Figure 2E). A gene closely associated with neural crest development, Sox10,^23^ was identified as one of the cell‐type specific transcriptional regulators of mesenchymal subset 4 (Figure 2E). Notably, cranial neural crest‐derived cells have been reported to regulate myogenic progenitors that differentiate into muscles; thus, *Sox10* may serve as a crucial potential target in the fusion process and development of cleft lip and palate (CL/P).^24^ *Barx1*, a gene uniquely expressed in mesenchymal subset 6, was found to be involved in maxillary morphogenesis.^25^ Notably, other cell‐type specific transcriptional regulator subtypes in mesenchymal subset 2, 4 and 6 may also perform important functions. Additionally, other mesenchymal subsets surrounding the fusion zone may be equally significant. For instance, Dlx1 and Dlx2 found in mesenchymal subset 1 have been reported to regulate interneuron differentiation between E10.5 and E12.5. Mice lacking Dlx1 and Dlx2 have been found to develop cleft palate.^26^ Furthermore, in mesenchymal subset 5, Sox9 emerged as a pivotal regulator of chondrogenesis^27^ (Figure S3A,B). Additionally, some cell‐type specific transcription factors and corresponding subpopulations of mesenchyme cells were co‐stained using immunofluorescence from E10.5 to E12.5 (Figure S4A).

Cell differentiation trajectory analysis was conducted on various mesenchymal cell subtypes to investigate the transitions among distinct subtypes and the underlying molecular mechanisms. For mesenchyme subsets 2, 4 and 6, the majority of cells from each subgroup were observed in the initial state. Conversely, for mesenchyme subsets 1, 3 and 5, the majority of cells were in the terminal state (Figure 2F). At E11.5, the subgroup cells were more evenly distributed (Figure S5A). Along the pseudotime, *Nfix* gene activation was highly significant. This gene has been previously shown to regulate the transition from embryonic to fetal myogenesis.^28^ Additionally, proteins associated with the extracellular matrix, such as COL1A1 and OGN, were significantly activated (Figure 2G).

### 3.2 Heterogeneity alterations of ectodermal cells throughout the process of upper lip and primary palate fusion

**FIGURE 3:**
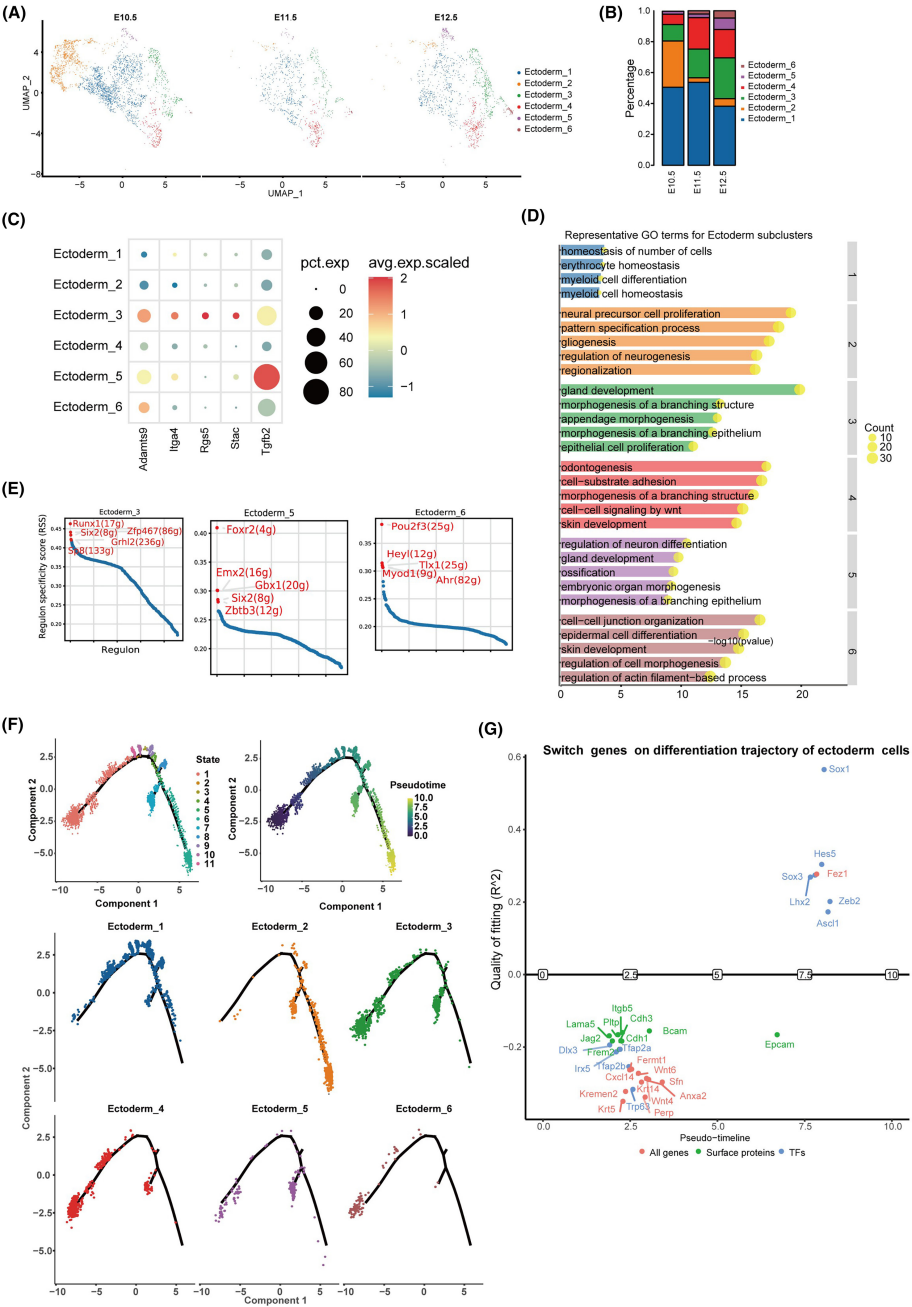
Heterogeneity changes of ectodermal cells. (A) UMAP plots displaying six subsets of ectodermal cells. (B) Bar diagrams presenting the ratio of subsets of ectodermal cells. (C) Dot plot showing the expression of selected marker genes for subgroups of ectodermal cells (upper); cell markers for sub‐ectoderm 6 cells. Circle size represents percentage of cells expressing the given marker gene; Red colour represents high expression while blue colour represents low expression. (D) The gene ontology analysis of subsets of ectodermal cells. (E) The top five regulons for ectoderm subset 3, 5 and 6 are highlighted in red and their expression changes from E10.5 to E12.5 were also shown. (F) Ectodermal cells were projected onto the different cell states (upper, left), and the pseudotime icons was shown (upper, right). Cellular differentiation trajectory for each subset of m ectodermal cells were shown in the lower. (G) Plot of switch genes on the differentiation trajectory of ectodermal cells. Genes above the horizontal line were switched‐on, while genes below the line were switched‐off.

Ectodermal cells, another crucial cell type involved in this fusion, could be further categorized into six subgroups (Figure 3A–C; Figure S2B; Table S4). The proportions of sub‐ectoderm 3 (Adamts9^+^, Itga4^+^, Rgs5^+^ and Stac^+^), sub‐ectoderm 5 (Tgfb2^+^) and sub‐ectoderm 6 (Krt5^+^) gradually increased from E10.5 to E12.5 (Figure 3B). Subgroups 3 and 5 were primarily associated with the morphogenesis of the branching epithelium, while subgroup 6 was primarily related to epithelial cell proliferation and skin development (Figure 3D). Spatial characterization of representative subgroups was revealed by immunofluorescence, which showed that parts of sub‐ectoderm 3 (Igfbp5^+^) and 5 (Dlx5^+^) were at the fusion zone at E11.5 (Figure S6). According to our previous RNAScope staining of krt5, sub‐ectoderm 6 (Krt5^+^) also existed at the fusion zone at E11.5.^11^ The *Six2* gene serves as a common transcriptional regulator for both ectodermal subgroups 3 and 5, which have been implicated in the development of the palate (Figure 3E; Table S5). Notably, a significant proportion of *Six2* null mice embryos, specifically 22%, exhibited cleft palate.^29^, ^30^ Upon further examination of ectodermal subgroup 6, it was determined to be equivalent to the periderm. This subgroup expressed canonical marker genes including *Sfn*, *Irf6*, *grh13*, *Arhgap29*, *Cldn3*, *Gabrp*, *Nebl* and *Krt5*, among others.^31^, ^32^, ^33^, ^34^, ^35^ The periderm has been reported to play a crucial role in preventing pathological epithelial adhesions during embryogenesis.^36^ Additionally, we identified a specific expression of *Ahr* within ectodermal subgroup 6. This expression has been associated with the development of cleft palate induced by 2,3,7,8‐tetrachlorodibenzo‐p‐dioxin^37^ (Figure 3E). Other transcriptional regulators expressed by these ectodermal subtypes may hold considerable significance. The ectoderm subsets surrounding the fusion zone also appear to be of utmost importance. For instance, *vax1*, identified in ectodermal subset 2, has been demonstrated to be linked with the development of cleft lip and palate (CL/P) in the Chinese population^38^ (Figure S3C,D). Within ectodermal subgroup 4, *Trp63* serves as the cell‐type specific transcriptional regulator. Notably, mutations in *Trp63* have been identified as one of the primary causes of cleft lip and palate.^39^ Additionally, some cell‐type specific transcription factors and corresponding subpopulations of ectoderm cells were co‐stained using immunofluorescence from E10.5 to E12.5 (Figure S4B).

Upon analysing the cell differentiation trajectory of the ectodermal cell subtypes, it was observed that ectoderm‐4 was primarily distributed at the initial state at E10.5, E11.5 and E12.5 (Figure S5B). Cells belonging to ectodermal subgroup 3 and 6 were primarily distributed at the initial state (Figure 3F). Cells of the main subgroup type, subgroup 1, were primarily distributed at the middle state. The genes *Sox1*, *Sox3* and *Hes5* exhibited significant activation along the pseudotime trajectory, as demonstrated in Figure 3G. Notably, *Hes5* plays a crucial role in Wnt‐3a‐induced neural differentiation.^40^ Conversely, switch genes such as *Tfap2a*, *Tfap2b* and *Sfn* were gradually inactivated and primarily associated with tube development and anchoring junctions along the pseudotime.^41^, ^42^

### 3.3 Mesenchymal and ectodermal cells possessed a marked different open chromatin profile for genes important for the fusion process

Utilizing our annotated scRNA‐seq data, we predicted scATAC‐seq cell types by referencing previous studies.^43^ A comparison between the predicted scATAC‐seq cell types and curated annotations confirmed the presence of all major cell types in both datasets. These findings also suggested that scATAC‐seq was comparable to scRNA‐seq in terms of cellular identity assignment, as depicted in Figure S7A,B. Therefore, multimodal scATAC‐seq and scRNA‐seq analysis were combined, significantly enhancing the identification of cellular heterogeneity.

**FIGURE 4:**
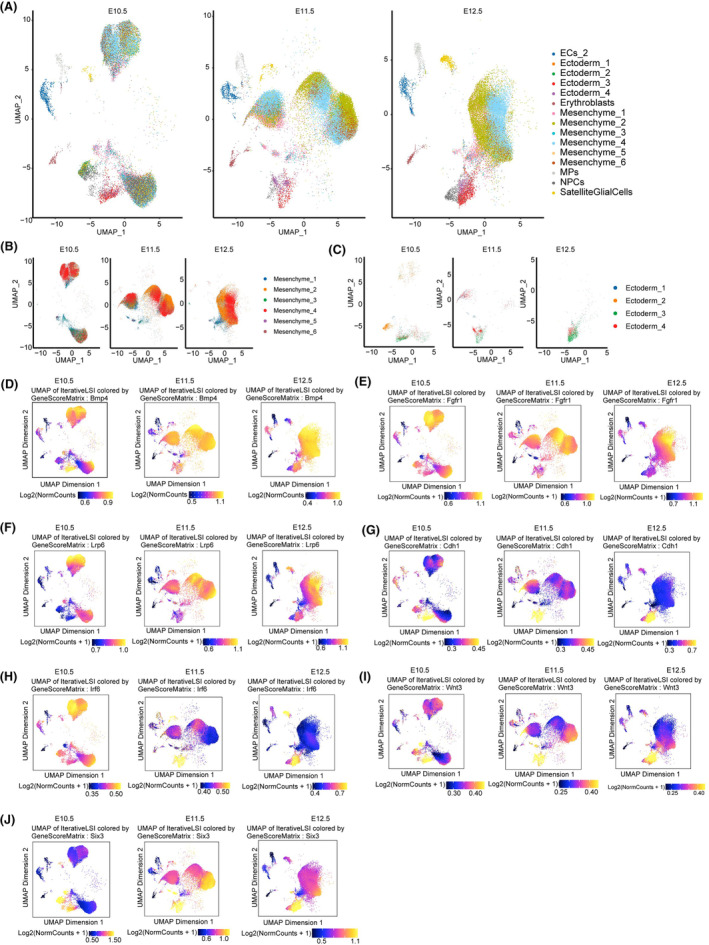
Single‐cell level chromatin accessibility analysis of target genes. (A–C) UMAP plots of scATAC‐seq data. (D–J) UMAP plots of chromatin accessibility of *Bmp4* (D), *Fgfr1* (E), *Lrp6* (F), *Cdh1* (G), *Irf6* (H), *Wnt3* (I) and *Six3* (J). Yellow colour represents high accessibility while blue colour represents low accessibility.

Genes with an open chromatin structure were found to be expressed, and a strong correlation was observed between highly expressed genes and increased chromatin openness.^44^, ^45^ We investigated the cell‐type specific chromatin accessibility of a series of pivotal genes involved in upper lip and primary palate development, based on the single‐cell chromatin accessibility landscape acquired using scATAC‐seq (Figure 4A). Mutations or abnormal expression of these genes have been associated with CL/P, a prevalent developmental malformation.^6^, ^12^, ^46^, ^47^, ^48^, ^49^ The differential accessibility of genes was observed between the mesenchymal and ectodermal cells, which play crucial roles in the fusion of the upper lip and primary palate. Specifically, *Fgfr1* (Figure 4A,B,E; Figure S8B) and *Lrp6* (Figure 4A,B,F; Figure S8C) exhibited greater chromatin openness in mesenchymal cells, whereas *Cdh1* (Figure 4A,C,G; Figure S8D), *Wnt3* (Figure 4A,C,I; Figure S8F) and *Six3* (Figure 4A,C,J; Figure S8G) were primarily expressed in ectodermal cells. Additionally, *Bmp4* (Figure 4A–D; Figure S8A) demonstrated increased chromatin openness in both cell types. Conversely, the chromatin openness of *Irf6* (Figure 4A–C,H; Figure S8E) decreased gradually in mesenchymal cells and increased gradually in ectodermal cells between E10.5 and E12.5. These findings provide a comprehensive atlas of dynamic chromatin accessibility for key genes involved in upper lip and primary palate fusion during this developmental period. Furthermore, they serve as a valuable resource for future studies on congenital defects at the single‐cell level.

### 3.4 Mesenchymal cells were the primary source of signal pathways throughout fusion

**FIGURE 5:**
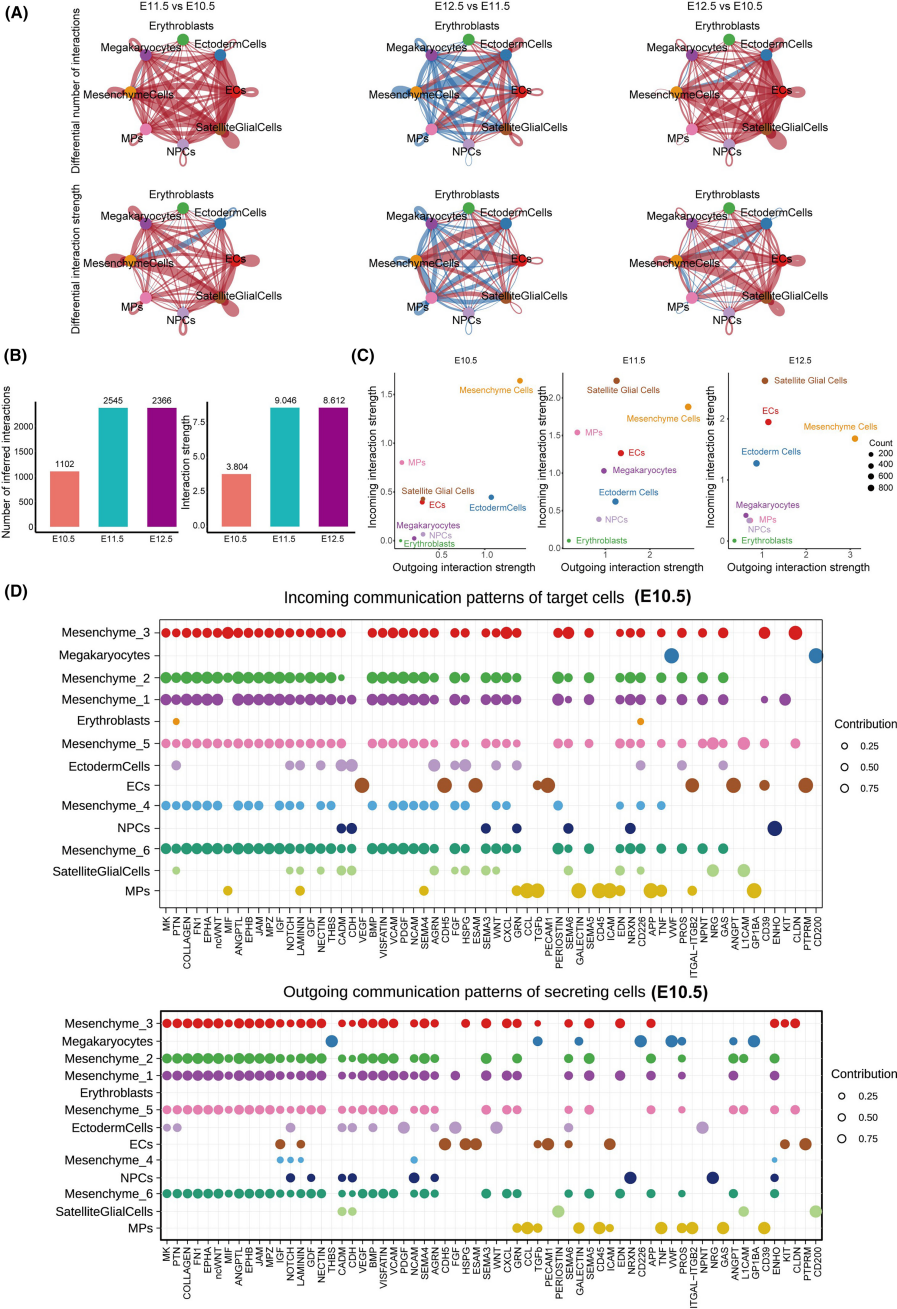
Cell–cell communication network among all cell types analysed with CellChat. (A) Circle plots present the differential number of interactions (upper panel) or interaction strength (lower panel) in the cellular communication network among E11.5 versus E10.5, E12.5 versus E11.5 and E12.5 versus E10.5 comparison groups, respectively. Red or blue edges indicate increased or decreased signalling among E11.5 versus E10.5, E12.5 versus E11.5 and E12.5 versus E10.5 comparison groups, respectively. (B) Bar graphs represent the number (left) or strength (right) of cellular interactions at E10.5, E11.5 and E12.5. (C) The distribution of cell clusters based on their incoming and outgoing signalling pattern counts. Y axis indicates incoming interaction strength, and X axis indicates outgoing interaction strength. (D) Dot plots showing the CellChat signalling in each mesenchyme cell subtypes at E10.5. The upper panel represents the incoming signalling patterns and the lower panel shows the outgoing signalling patterns. Colours of dots correspond to the relevant cell types. The bubble size indicates the degrees of expression weight value of signalling molecules or receptors.

The CellPhoneDB algorithm was utilized to investigate the intercellular communications among distinct cell types within the lambdoidal junction tissue. The results indicated that E11.5 exhibited the highest number of inferred interactions and the strongest interaction strength, as evident in Figure 5A,B. This finding suggests that E11.5 represents the most crucial and intricate stage during the development of the upper lip and primary palate. Specifically, mesenchymal cells demonstrated the highest outgoing interaction strength at E10.5, E11.5 and E12.5, serving as the primary source of detected signals, as shown in Figure 5C.

Subsequently, a detailed evaluation was conducted on the cellular interactions between mesenchymal cell subgroups and other cell types. A range of potential signals were identified that impact the fusion process of the upper lip and primary palate. Notably, the communication patterns among all mesenchymal cell subgroups were similar, and they functioned as the primary source and receiver of intercellular signals between E10.5 and E12.5, as depicted in Figure 5D and Figure S9A,B. In terms of signal reception, mesenchymal cells received numerous signals, whereas ectodermal cells were primarily influenced by a limited number of signals throughout the fusion process, including RELN, SEMA6, HSPG, CADM, OCLN and AGRN signals.

### 3.5 Important signalling pathways involved in the fusion process experienced distinct dynamic changes throughout the fusion process

**FIGURE 6:**
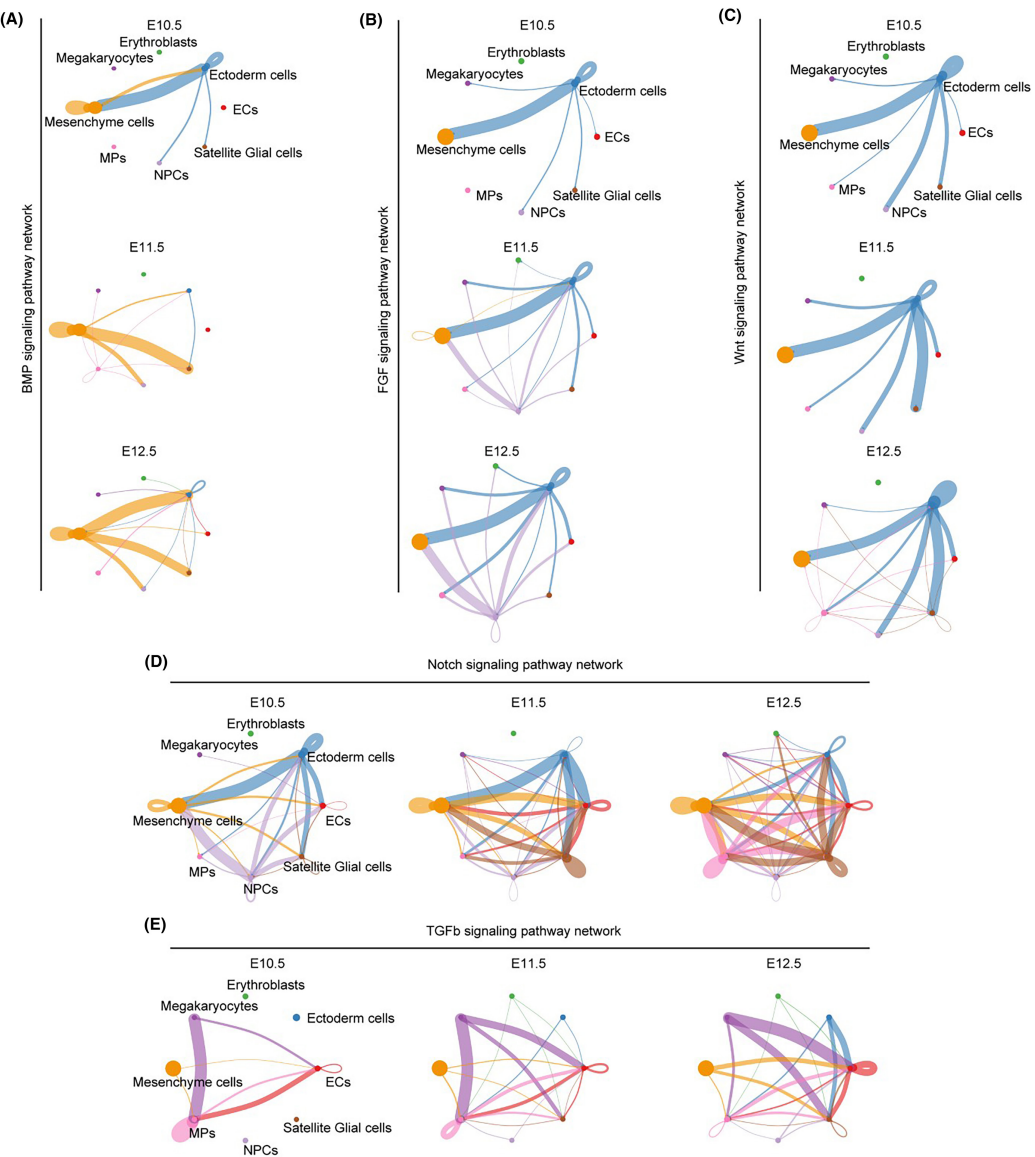
Dynamic alterations of important signals during the formation of upper lip and primary palate. (A–E) Circle plots presenting selected differential signalling networks. Line colours were consistent to colours of signalling producing cell type. The width of edge indicates the communication probability.

As BMP, WNT, FGF, TGFβ and NOTCH signalling pathways play crucial roles in the development of the upper lip and primary palate, we conducted a detailed analysis of the alterations in cellular interactions within these pathways throughout the fusion process. Our findings, based on observations from E10.5 to E12.5, revealed that BMP signal was primarily generated by mesenchymal cells (Figure 6A), WNT and FGF signals were primarily produced by ectodermal cells (Figure 6B,C), TGFβ signal was primarily generated by megakaryocytes (Figure 6G), while NOTCH signal could be produced by several cell types (Figure 6E). Specific to the different subpopulations, the WNT signal was mainly produced by sub‐ectoderm 4, the FGF signal was mainly produced sub‐ectoderm 3 (Figure S10). Notably, sub‐ectoderm 6 is the main receiving subgroup of NOTCH signalling (Figure S10). In terms of interaction strength, a robust Bmp signalling was observed between ectodermal and mesenchymal cells at E10.5 and E12.5. However, this interaction exhibited a noticeable weakening at E11.5 (Figure 6A). Notably, the strength of FGF and WNT signalling between these two cell types remained consistently high throughout the fusion process, indicating their significant influence on the entire fusion process (Figure 6B,C). Additionally, a strong NOTCH signalling was also observed between ectodermal and mesenchymal cells at both E10.5 and E11.5 (Figure 6D). Previous studies have reported that TGFβ signalling plays a pivotal role in controlling soft palate muscle development by regulating epithelial–mesenchymal interactions.^50^ Consistent with these reports, our findings also suggest a gradual increase in interactions between endothelial and mesenchymal cells for TGFβ signalling (Figure 6E). Furthermore, our analysis revealed the involvement of several novel potential signals, including the PDGF, GDF and PTN signalling pathways, in the development of the upper lip and primary palate. Collectively, our findings suggest the existence of a comprehensive and dynamic signalling network that underlies the development of the upper lip and primary palate.

### 3.6 Upstream regulators of important signalling pathways involved in the fusion process were predicted

**FIGURE 7:**
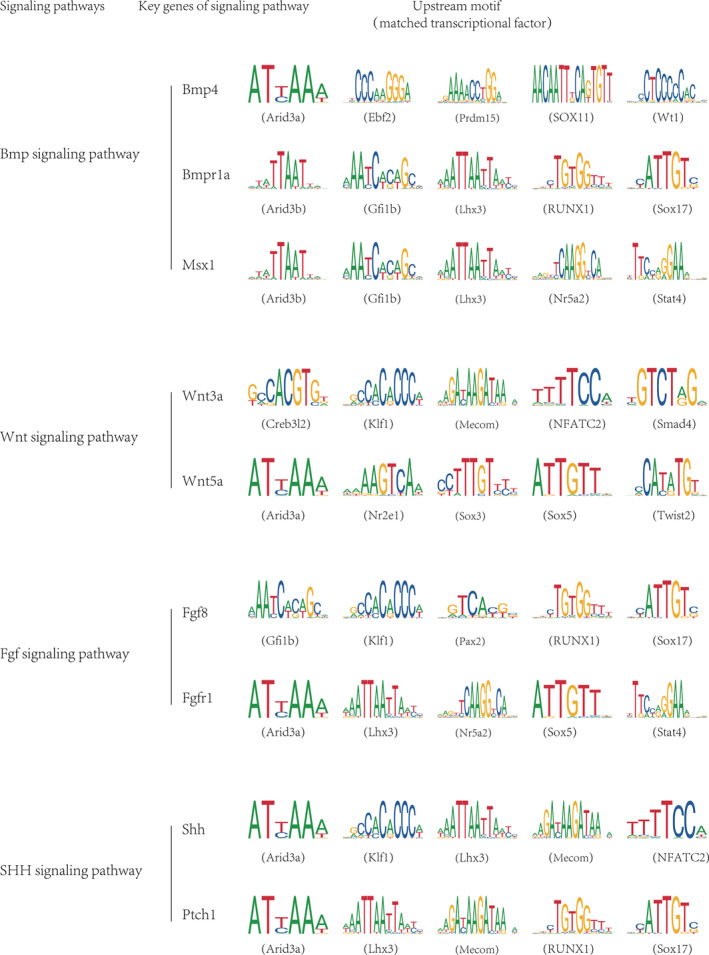
Corresponding motifs characterized as significant in the scATAC‐seq analysis for pivotal genes of important signals are shown.

Motif analysis was conducted using the JASPAR 2022 database^51^ to investigate potential novel upstream regulators of key genes involved in critical signalling pathways, specifically BMP, WNT, FGF and SHH, which are known to play a pivotal role in upper lip and primary palate development^52^, ^53^ (Figure 7, Table S6). For the BMP signalling pathway, several predicted upstream regulators were identified for *Bmp4*, *Bmpr1a* and *Msx1*, including *Arid3a*, *Ebf2*, *Prdm15*, *Sox11, Wt1*, *Arid3b*, *Gfi1b*, *Lhx3*, *Runx1*, *Sox17*, *Nr5a2* and *Stat4*. Similarly, for the WNT pathway, upstream regulators were predicted for *Wnt3a* and *wnt5a*, including *Creb3l2*, *Klf1*, *Mecom*, *Nfatc2*, *Smad4*, *Arid3a*, *Nr2e1*, *Sox3*, *Sox5d* and *Twist2*. For the FGF signalling pathway, upstream regulators were predicted for *Fgf8* and *Fgfr1*, including *Gfi1b*, *Klf1*, *Pax2*, *Runx1, Sox17*, *Sox5*, *Lhx3*, *Arid3a*, *Nr5a2* and *Stat4*. Moreover, for the SHH signalling pathway, upstream transcriptional factors were predicted for *Shh* and *Ptch1*, including *Arid3a*, *Klf1*, *Lhx3*, *Mecomd*, *Nfatc2*, *Runx1* and *Sox17*. Certain predicted upstream regulators were found to be shared; these included *Mecom*, *Lhx3* and *Sox17*. Transcriptional regulators play a crucial role in the functioning of signalling pathways involved in upper lip and primary palate development. While Runx1 is known to be essential for anterior‐specific palatal fusion,^54^ most of the identified predicted regulons have not been reported previously. These findings suggest the presence of novel regulators that may play a significant role in the fusion process. Overall, our results provide a comprehensive list of predicted upstream regulators of key genes involved in critical signalling pathways for normal upper lip and primary palate development.

## 4 DISCUSSION

Our analysis of single‐cell data reveals that mesenchymal cells constitute the predominant cell type, followed by ectodermal cells, in the lambdoidal junction tissues during the development of the upper lip and primary palate. These observations align with the findings of a prior study.^8^ Despite the observed variations among MxP, LNPs and MNP, immunostaining demonstrated the presence of subgroups of major cell types, such as mesenchymal and ectodermal cells, distributed in a dispersed manner throughout these regions. The fusion process of the upper lip and primary palate involves epithelial apoptosis and migration, epithelial‐to‐mesenchymal transition and mesenchymal proliferation. Upon completion of fusion, mesenchymal cells differentiate into skeletal, muscular and connective tissue cells. Our study, which encompassed multiple time points, enabled the precise characterization of heterogeneous alterations in distinct cell types throughout the fusion process. Previous studies have demonstrated the presence of mesenchymal cells expressing Cxxc4, Rerg and Aldh1a2 in the fusion zone, while Tgfb2 marks the basal cells in this area.^8^ In our study, sub‐mesenchyme 2, 4 and 6 expressed these markers. Additionally, ectodermal cells expressing Adamts9, Itga4, Rgs5 and Stac were also found in the fusion zone.^8^ Correspondingly, in our study, sub‐ectoderm 3, 5 and 6 expressed these markers. As for the three sub‐ectodermal cell populations, although the fusion zone may have disappeared by the time the fusion is completed, they are still present in the fusion tissues on the buccal side and on the surface of the lateral nasal protrusion, which suggests that the cells in the fusion zone are also present in the rest of the three protrusions, and are not only specific to the fusion zone. This can also be confirmed by our previous RNAScope staining of krt5, also a marker for ectodermal cell subpopulation 6.^11^ Thus, this study may also provide inspiration for studies of orofacial development.

Given that transcriptional factors play a pivotal role in regulating cell fates and are crucial for tissue and organ formation during embryonic development,^55^ the identity of cell‐type specific transcriptional factors involved in the fusion process remained enigmatic. Our findings provide a more detailed characterization of numerous cell‐type specific transcriptional regulators, including *Sox10*, *Barx1*, *Six2*, *Trp63*, *Ahr* and others. Notably, certain genes among these regulators have been previously associated with cleft lip and palate (CL/P). For instance, the Sox10 gene, present in mesenchyme subset 4, has a close association with neural crest development.^23^ This neural crest‐derived cells have been reported to regulate the differentiation of myogenic progenitors into muscles of the soft palate, indicating the significance of *Sox10* in upper lip and primary palate development.^24^ Additionally, *Six2*, a transcriptional regulator shared by ectodermal subgroups 3 and 5, has been implicated in palate development. Notably, 22% of *Six2* null mouse embryos exhibited cleft palate.^29^, ^30^ Furthermore, mutations in the cell‐type specific transcriptional regulator *Trp63* of ectodermal subgroup 4 have been demonstrated to cause CL/P.^39^ We also performed immunofluorescence co‐staining for specific transcription factors and corresponding subpopulations. Although subgroups of major cell types were dispersedly existed among these three prominences, there existed some differences for the distribution of transcriptional factors among three prominences. For example, mesenchyme 4 (col2a1^+^, Igfbp5^+^) specific transcriptional factor, Sox10, was mainly expressed in Mxp. However, for more genes, like Runx1, Rab14, Trp63 and Twist1 were dispersedly expressed across three prominences. However, the roles of other regulators that exhibit strong phenotypes in other tissues or organs have not been systematically examined in the context of upper lip and primary palate formation. These cell‐type specific transcriptional regulators may not only maintain the distinct subtypes of cells but also directly or indirectly influence upper lip and primary palate fusion. Abnormal expression or mutations of these regulators could result in developmental abnormalities of the lip and palate.

Cellular heterogeneity is a widespread phenomenon throughout embryonic development. However, the specific molecules that drive heterogeneous changes in primary cell subgroups during the fusion process remain poorly understood. Previous research has demonstrated the involvement of cellular migration, differentiation and apoptosis in the fusion of the upper lip and primary palate.^56^, ^57^ In our study, we characterized a series of switch genes involved in the apoptotic process, including *Trp63*, *Sfn*, *Tfap2a*, *Tfap2b* and *Perp*.^58^, ^59^, ^60^, ^61^ Notably, mesenchymal subgroups exhibited a highly activated myogenesis‐associated gene, *Nfix*,^28^ while extracellular matrix‐related proteins such as COL1A1 and OGN were strongly expressed during the differentiation pseudotime. In the ectodermal subgroups, a series of novel upregulated switch genes were found to be associated with neural differentiation pseudotime, including *Sox1*, *Sox3* and *Hes5*.^40^, ^62^ Additionally, downregulated switch genes in ectodermal cells were primarily involved in epithelial development (*Tfap2a*, *Tfap2b*, *Sfn*, *Igfbp5*, *Dlx3*, *Krt5* and *Wnt4*) and anchoring junctions (*Anxa2*, *Cdh3*, *Epcam*, *Cdh1* and *Perp*) along the pseudotime. These findings suggest that dynamic alterations in the expression of switch genes involved in cellular transitions may facilitate upper lip and primary palate fusion and could serve as potential candidates for future studies.

Given that related chromatin regions are known to open prior to gene expression,^44^, ^63^ we also investigated changes in cell‐type specific chromatin accessibility of pivotal genes throughout the fusion process, in addition to cellular and molecular alterations. The genes studied encompassed *Bmp4*,^46^ *Cdh1*,^64^ *Fgfr1*,^65^ *Wnt3*,^66^ *Irf6*,^67^ *Lrp6* ^12^ and *Six3*.^68^ It was noteworthy that scATAC‐seq analysis predicted mesenchymal cells as the primary cell type expressing *Fgfr1* and *Lrp6*, while ectodermal cells were the primary cell type expressing *Cdh1*, *Wnt3* and *Six3*. Both mesenchymal and ectodermal cells were predicted to express *Bmp4* at high levels. Notably, the predicted expression levels of *Irf6* decreased gradually in mesenchymal cells but increased progressively in ectodermal cells between E10.5 and E12.5, indicating functional switches between these two cell types. Mice with an Irf6 deficiency exhibit a thickened and disorganized epithelium characterized by rounded, undifferentiated‐looking cells, diminished skin barrier function and ectopic adhesions affecting tissues such as the oral cavity and oesophagus.

Tissue and organ development require precise coordination of multiple signalling pathways.^69^ Consistent with previous findings,^8^ our study underscores the significance and complexity of the E11.5 stage during upper lip and primary palate fusion. Our findings demonstrated that mesenchymal cells serve as the primary source and recipient of intercellular signals. Furthermore, our findings elucidated the intricate signal patterns among distinct cell types of lambdoidal junction tissues throughout the fusion process. Specifically, BMP signal was primarily produced by mesenchymal cells, while WNT and FGF signals were primarily produced by ectodermal cells. Strong BMP signalling was observed between ectodermal and mesenchymal cells at E10.5 and E12.5, but weakened at E11.5. By contrast, the strength of FGF and WNT signalling between ectodermal and mesenchymal cells remained high and unchanged throughout the fusion process. In terms of signal reception, mesenchymal cells received numerous signals, whereas ectodermal cells were primarily affected by a limited number of signals throughout the fusion process, including RELN, SEMA6, HSPG, CADM, OCLN and AGRN signals. Notably, sub‐ectoderm 6 is the main receiving subgroup of NOTCH signalling, a known signal involved in the fusion process. Additionally, other pathways that play crucial roles in tissue fusion and development were also identified in our study. Other signalling pathways exhibiting notable alterations, including the FGF, PDGF, GDF and PTN pathways, may play a crucial role in the formation of the upper lip and primary palate. This study also delved into the upstream transcriptional regulators of genes linked to less well‐understood pathways such as the BMP, WNT, FGF and SHH pathways. We identified shared upstream transcriptional regulators among these signalling pathways, including *Arid3a*, *Runx1* and *Mecom*. Among the newly identified motif‐associated transcription factors, *Arid3a* is vital for early embryonic survival^70^; while *Sox11*, *Ebf2* and *Wt1* are all involved in nervous system development.^71^, ^72^, ^73^ *Runx1*, identified as a predicted upstream regulator of *Bmpr1a*, is essential for anterior‐specific palatal fusion and chondrogenic differentiation.^54^, ^74^ *Nr5a2*, a pluripotency transcriptional factor, may promote cell type conversion,^75^ while *Mecom* and *Sox5* regulate endothelial cell and chondrogenic differentiation,^76^ respectively. The *Nfatc2* gene triggers the production of a specific myosin heavy chain isoform during embryogenesis,^77^ and *Twist2* is a key driver of epithelial‐to‐mesenchymal transition by inducing the expression of associated genes.^78^ Alterations in the activities of these transcriptional regulators can influence the execution of related signalling pathways, potentially leading to developmental changes or abnormalities. Our findings offer valuable insights into the upstream regulators of crucial signalling pathways and constitute a significant resource for studying their impact on upper lip and primary palate fusion.

In summary, our study evaluated multiple time points, revealing dynamic transcriptomic and epigenetic alterations among crucial cell types involved in the fusion of the upper lip and primary palate. Additionally, we identified novel transcriptional factors, genes and signals. Future analysis of integrative scRNA‐seq and scATAC‐seq datasets, alongside molecular and cellular alterations in animal models, could offer valuable insights into the normal development and associated abnormalities of the upper lip and primary palate.

## AUTHOR CONTRIBUTIONS

**Sini Cai:** Conceptualization (equal); data curation (equal); methodology (equal); software (equal); validation (equal); writing – original draft (equal). **Ningbei Yin:** Funding acquisition (equal); project administration (equal); supervision (equal); writing – original draft (equal); writing – review and editing (equal).

## FUNDING INFORMATION

This work was funded by CAMS Innovation Fund for Medical Sciences(2021‐I2M‐1‐052).

## CONFLICT OF INTEREST STATEMENT

The authors have no conflicts of interest to declare.

## Supporting information


Appendix S1.

## Data Availability

Datasets can be accessed at https://ngdc.cncb.ac.cn/gsa/s/owC7VygP, hosted at Genome Sequence Archive.
