# Supplementary material for: Single‐cell transcriptome and chromatin accessibility mapping of upper lip and primary palate fusion
Source: J Cell Mol Med. 2024 Oct 11;28(19):e70128. doi: 10.1111/jcmm.70128 (PMC11467802; doi:10.1111/jcmm.70128)
Supplement: Supplementary file 1 — Appendix S1. [file JCMM-28-e70128-s001.zip › Supplementary Information.pdf]

Supplementary Figures

Supplementary figure 1

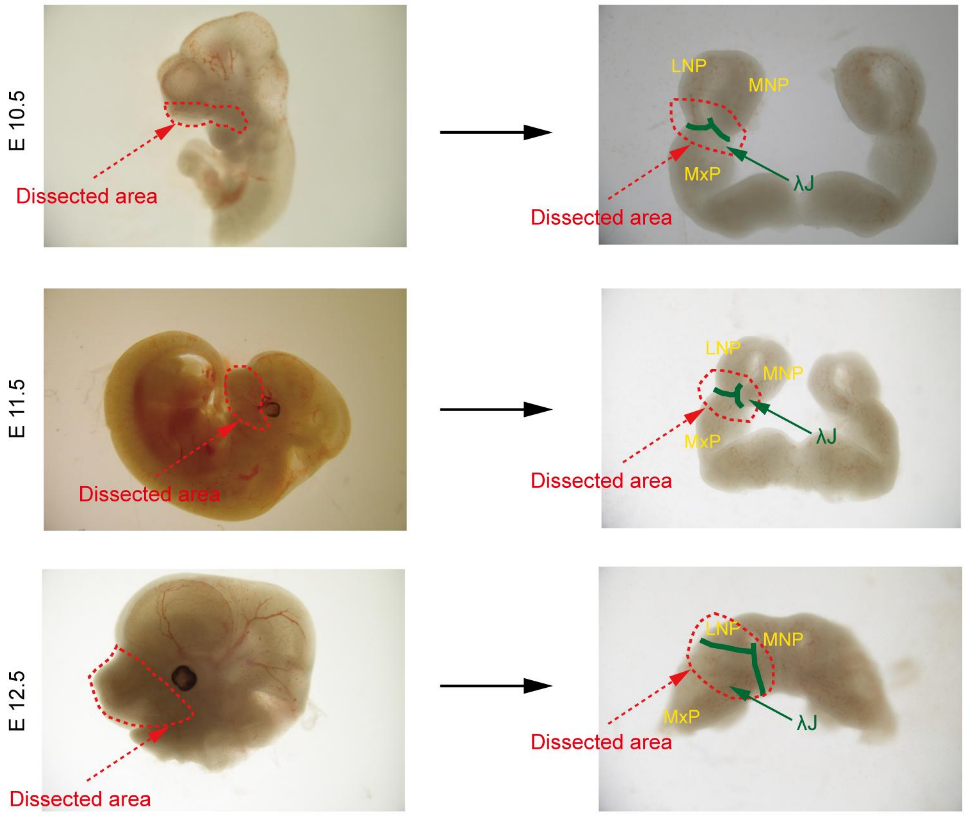

Supplementary figure 1. Schematic representation of E10.5, E11.5 and E12.5 upper mouse face with landmarks pertinent to the scATAC-seq and scRNA-seq analysis. LNP, lateral nasal prominence; MNP, medial nasal prominence; MxP, maxillary prominence; λJ, lambdoid junction.

Supplementary figure 2

A

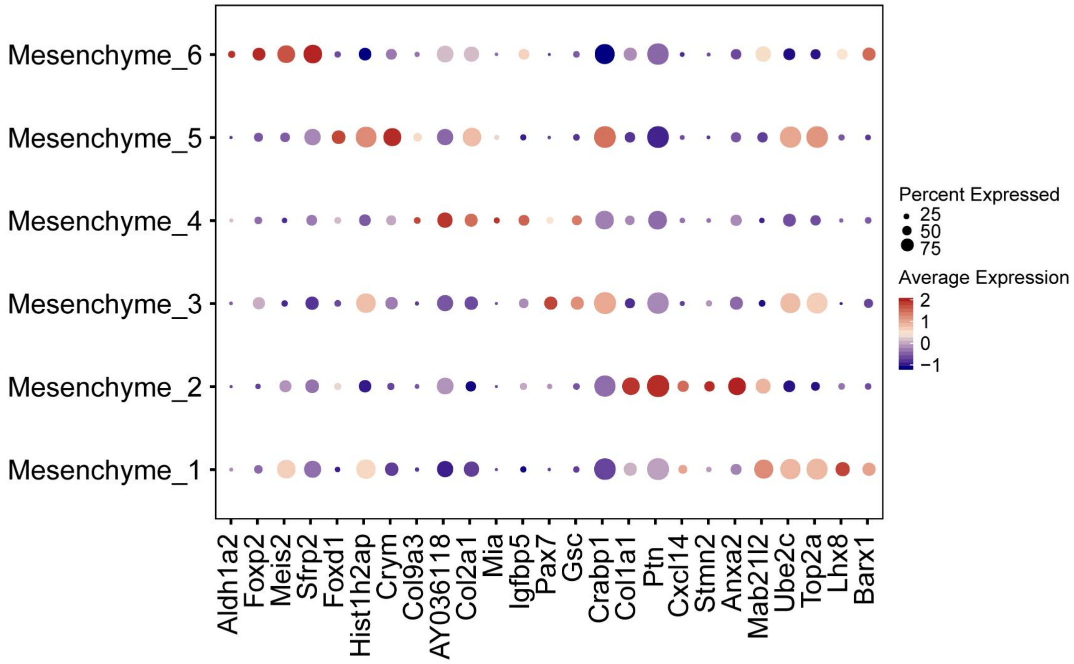

B

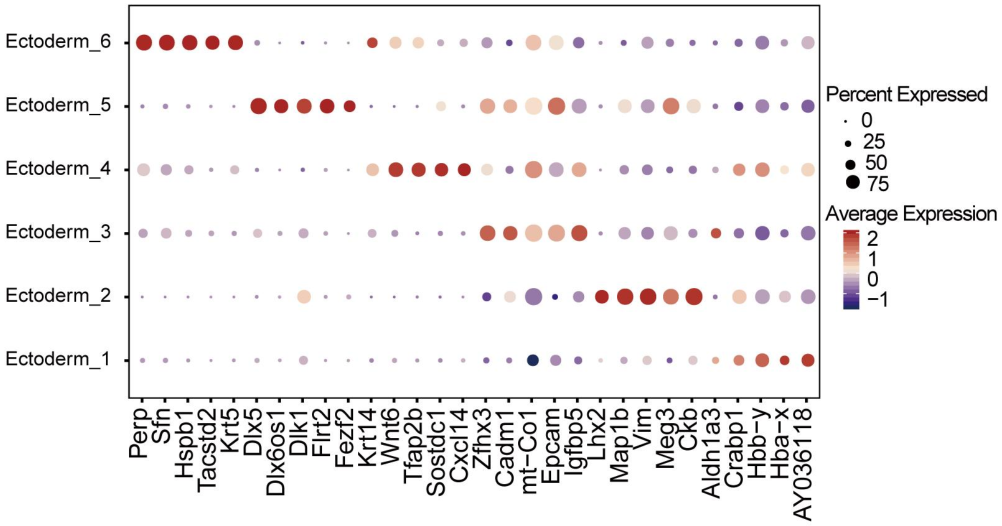

Supplementary figure 2. (A) Dot plot showing the expression of top 5 marker genes for subgroups of mesenchymal cells. (B) Dot plot showing the expression of top 5 marker genes for subgroups of ectodermal cells. Circle size represents percentage of cells expressing the given marker gene; Red color represents high expression while blue color represents low expression.

Supplementary figure 3

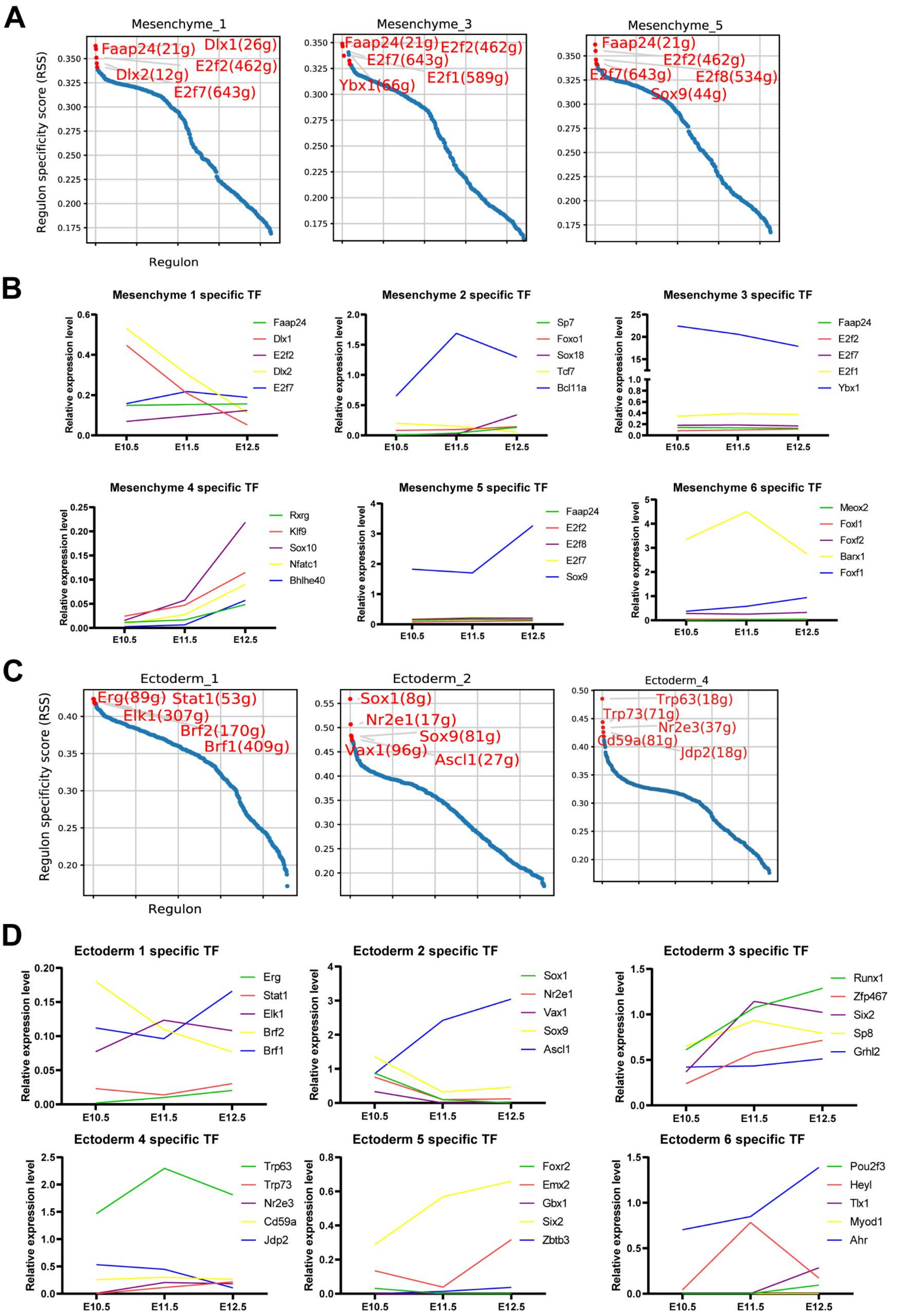

Supplementary figure 3. Transcription factor regulatory network analysis. (A) Regulon specificity score for mesenchyme subset 1， 3 and 5. The top five highest scoring regulons in each cell subtypes are highlighted in red. (B) The relative expression changes of the top five highest scoring regulons in each cell subtype of mesenchyme from E10.5 to E12.5 were shown. (C) Regulon specificity score for ectoderm subset 1，2 and 4. The top five regulons in each cell subtypes are highlighted in red. pySCENIC provides standard functionality to calculate the scores. (D) The relative expression changes of the top five highest scoring regulons in each cell subtype of mesenchyme from E10.5 to E12.5 were shown.

Supplementary figure 4

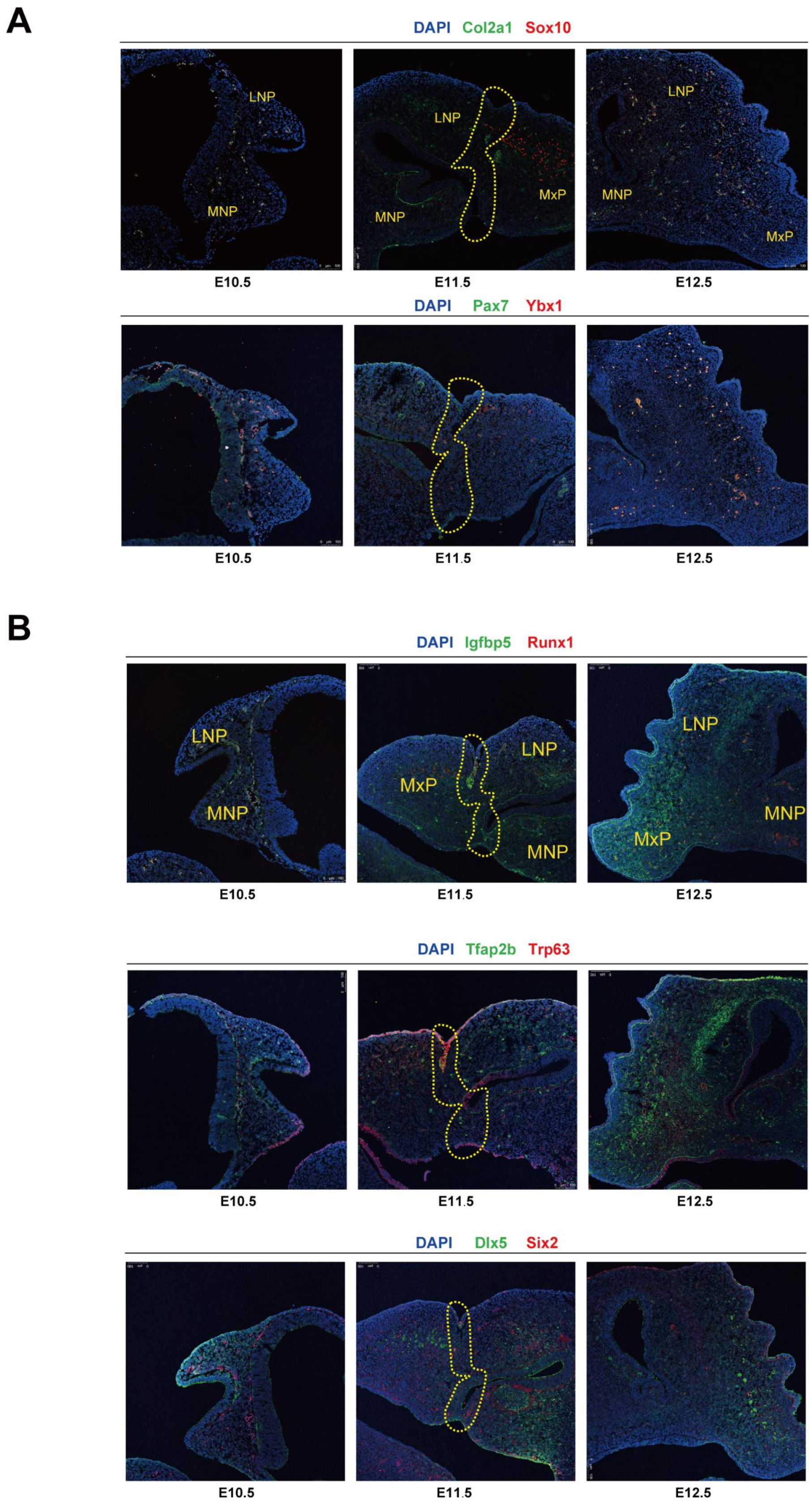

Supplementary figure 4. Immunofluorescence co-staining images of marker gene of cell subsets

(Col2a1is a cell marker of mesenchyme_4; Pax7 is a cell marker of mesenchyme_3; Igfbp5 a cell marker of ectoderm_3; Tfap2b is a cell marker of ectoderm_4; Dlx5 a cell marker of ectoderm_5) and cell subgroup-specific regulatory genes from E10.5 to E12.5. Marker genes of cell subtype, green; cell subgroup-specific regulatory genes, red; nucleus, blue. MxP: the maxillary prominence; LNP: the lateral nasal; MNP: the medial nasal. The fusion junction was marked with a yellow dashed line.

Supplementary figure 5

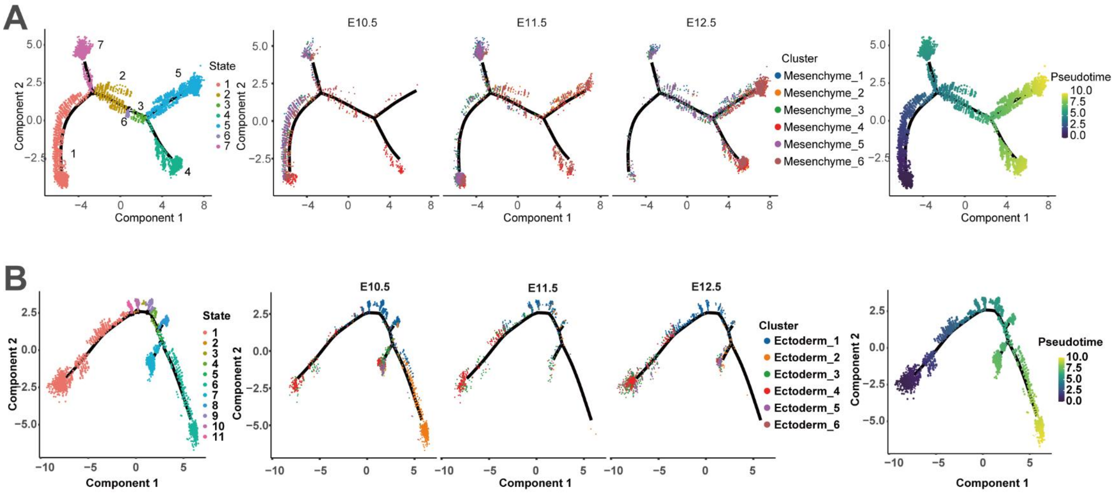

Supplementary figure 5. Reconstruction of cellular differentiation trajectory from E10.5 to E12.5. (A-B) Mesenchyme (A) and ectoderm cells (B) were projected onto the different cell states (left), And cellular distribution at E10.5, E11.5 and E12.5 was shown (middle), and the pseudotime icons was shown in the right.

Supplementary figure 6

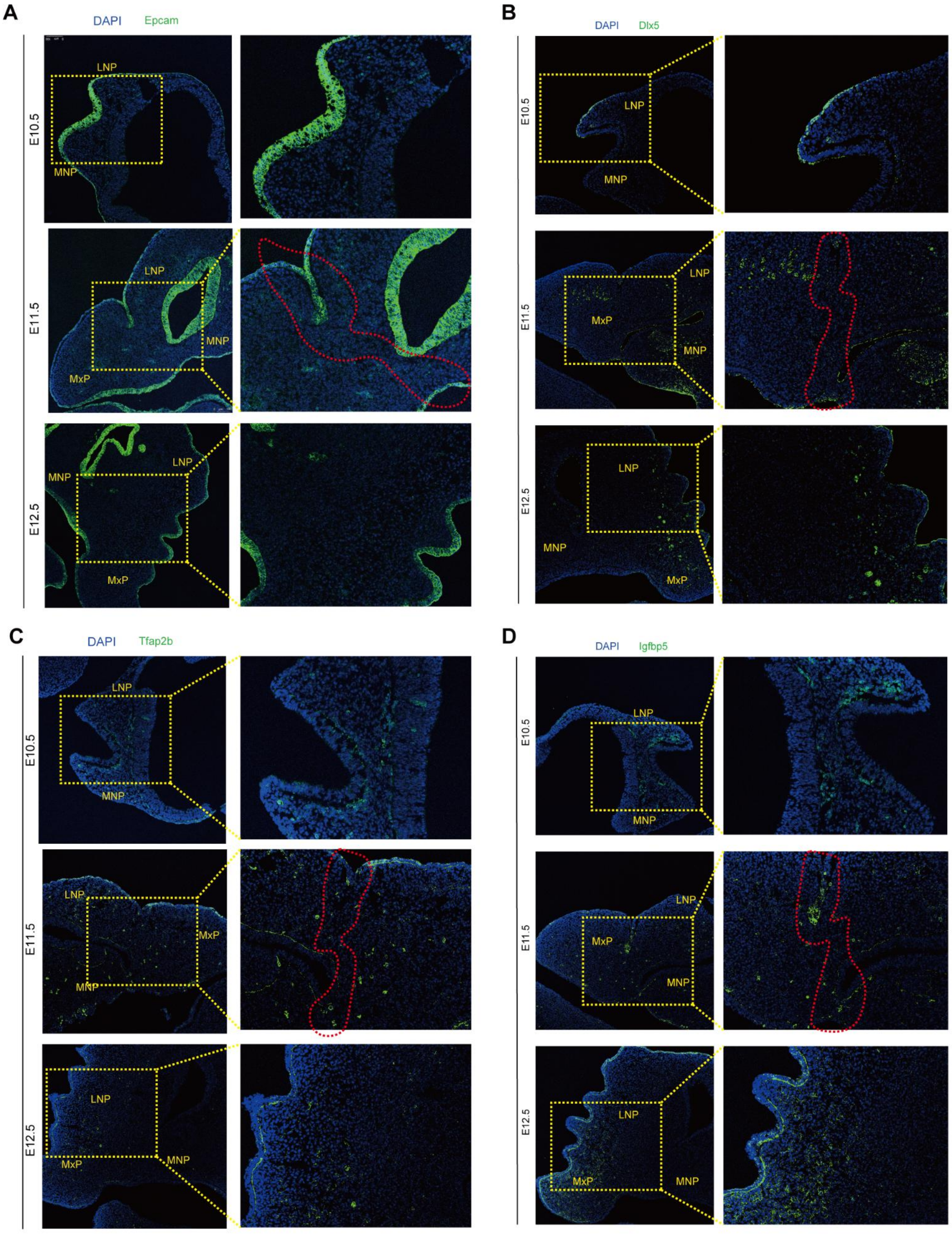

Supplementary figure 6. Immunofluorescence staining images of marker gene of representative ectoderm cell subsets from E10.5 to E12.5. Marker genes of cell subtype, green; cell subgroup-specific regulatory genes, red; nucleus, blue. MxP: the maxillary prominence; LNP: the lateral nasal; MNP: the medial nasal. The fusion junction was marked with a red dashed line.

Supplementary figure 7

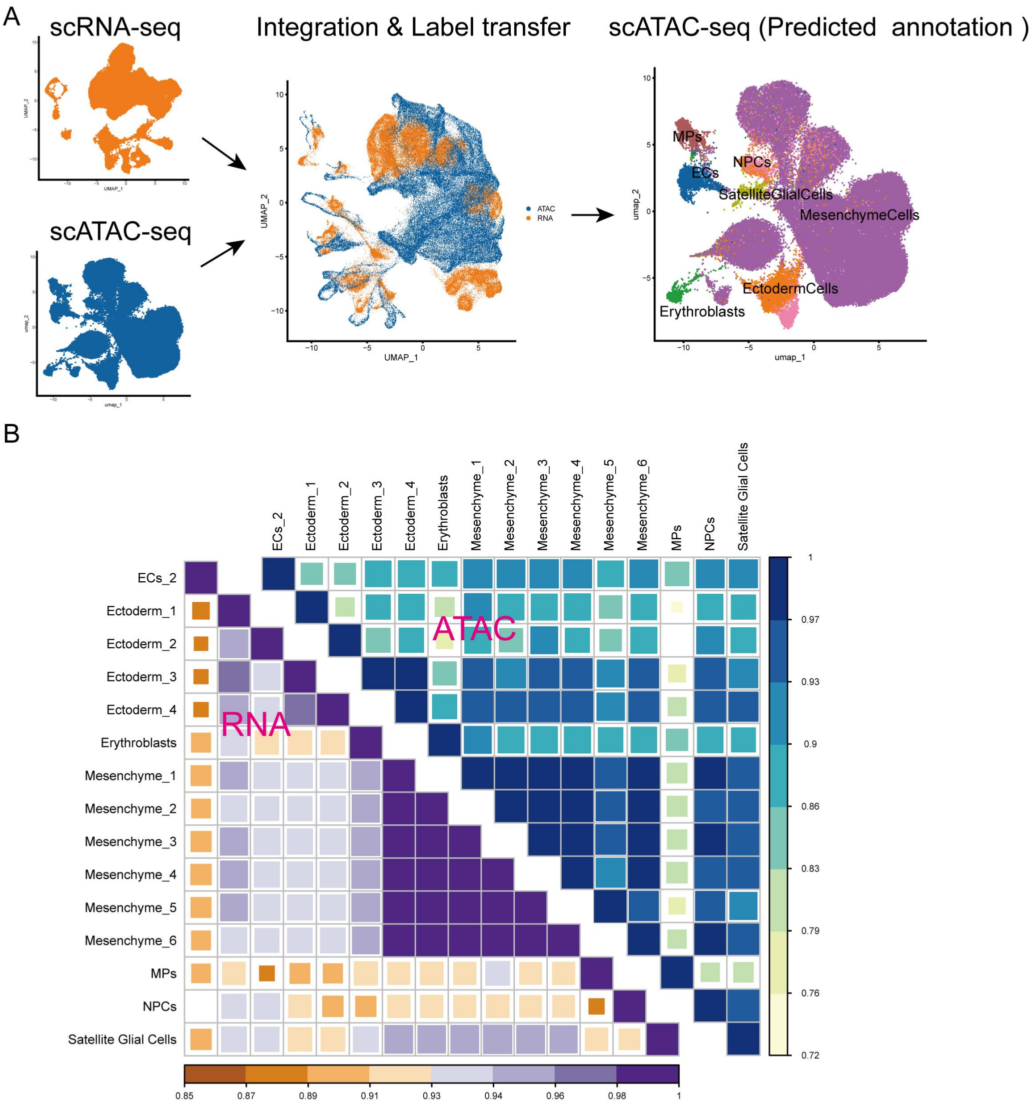

Supplementary figure 7. Combination of scRNA-Seq and scATAC-Seq Data. (A) Multi-omics integration strategy for scATAC-seq data processing. The scATAC-seq data was subjected to integration, label transfer and cell-type assignment. (B) Spearman correlation analysis on scATAC-seq and scRNA-seq. The average peak counts of each cluster were used to calculate the scATAC-seq correlation. The average gene expressions of each cluster were used to calculate the scRNA-seq correlation.

Supplementary figure 8

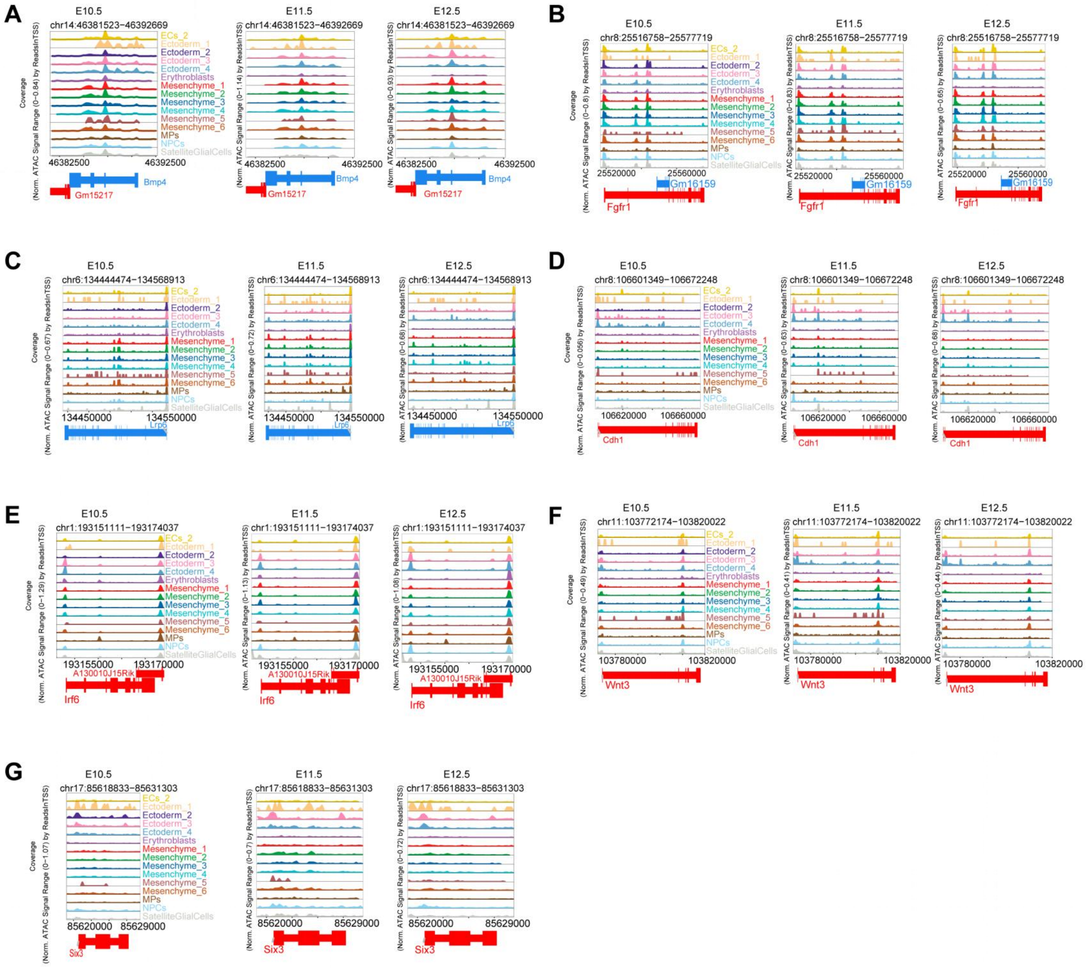

Supplementary figure 8. Normalized ATAC-seq profiles of endothelial cell type 2, ectoderm-1, ectoderm-2, ectoderm-3, ectoderm-4, erythroblast, mesenchyme-1, mesenchyme-2, mesenchyme-3, mesenchyme-4, mesenchyme-5, mesenchyme-6, mononuclear phagocytes (MPs), nuclear progenitor cells (NPCs) and satellite glial cells at the *Bmp4* (A), *Fgfr1* (B), *Lrp6* (C), *Cdh1* (D), *Irf6* (E), *Wnt3* (F) and *Six3* (G) gene loci.

Supplementary figure 9

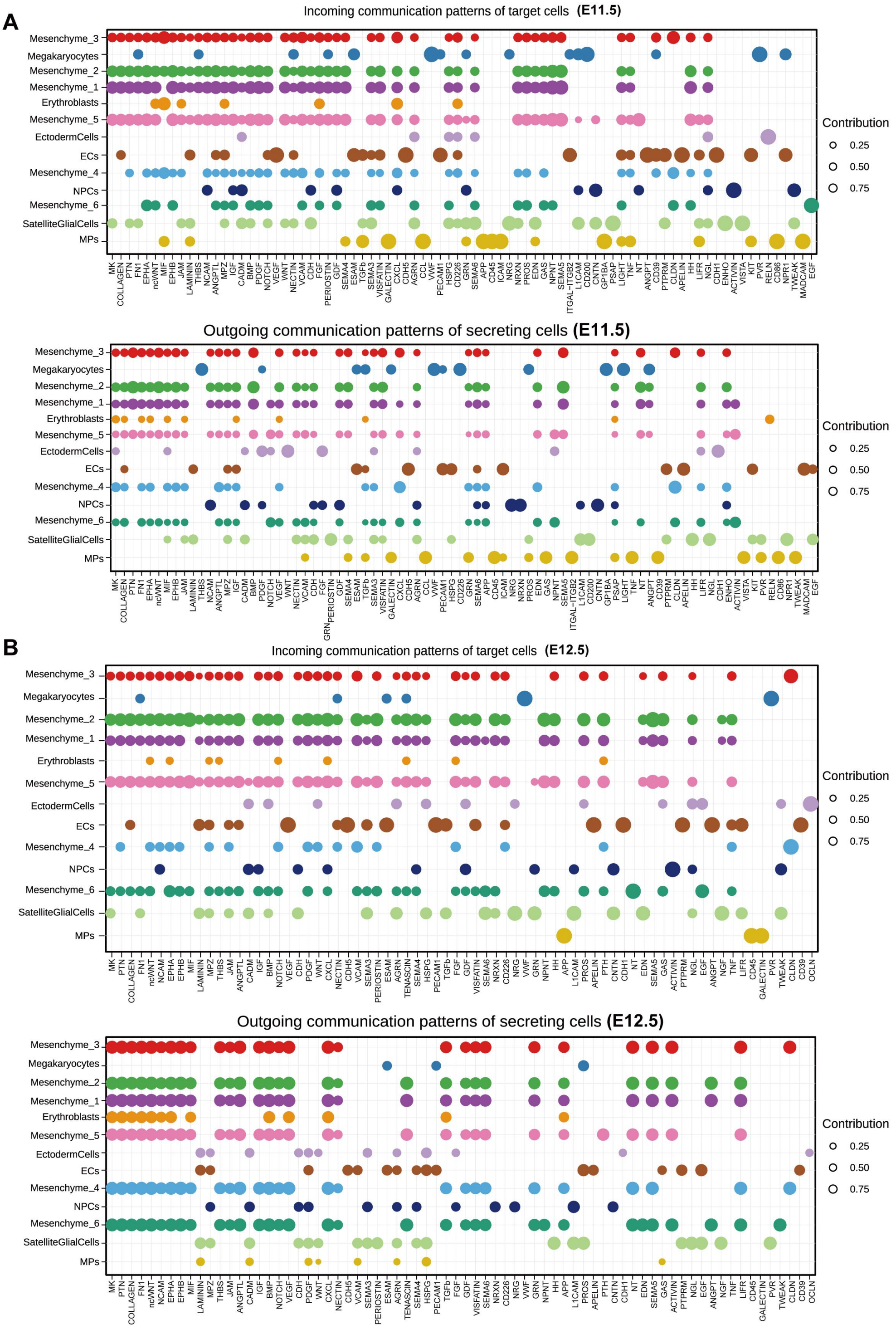

Supplementary figure 9. CellChat signalling in each mesenchyme cell subtypes at E11.5 and E12.5. (A-B) Dot plots showing the CellChat signalling in each mesenchyme cell subtypes at E11.5 (A) and E12.5 (B). The upper panels of (A) and (B) represent the incoming signalling patterns and the lower panels of (A) and (B) show the outgoing signalling patterns. Colors of dots

correspond to the relevant cell types. The bubble size indicates the degrees of expression weight value of signaling molecules or receptors.

Supplementary figure 10

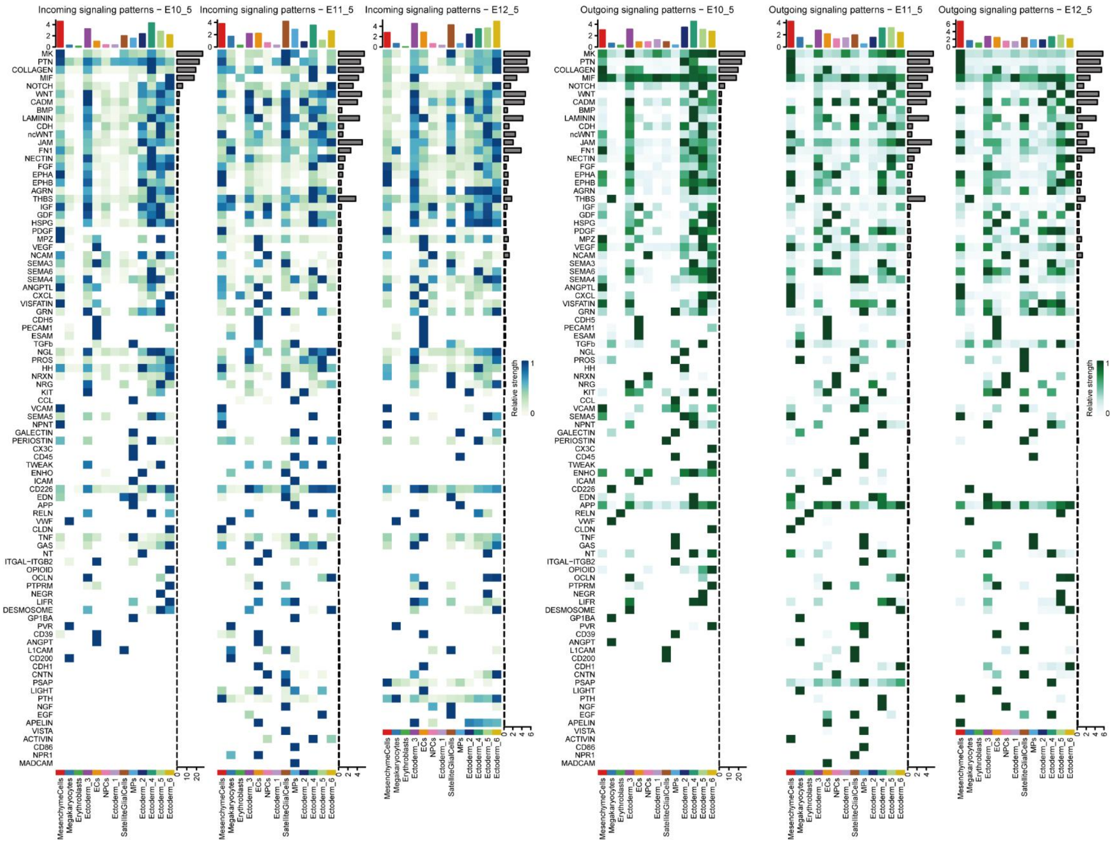

Supplementary figure 10. CellChat signalling in each ectoderm cell subtypes at E10.5, E11.5 and E12.5. The left three panels of represent the incoming signalling patterns and the right three panels show the outgoing signalling patterns. Colors of dots correspond to the relevant cell types. The bubble size indicates the degrees of expression weight value of signaling molecules or receptors.

Supplementary tables 1 to 6:

Supplementary table 1: Differentially expressed genes in main cell types.

Supplementary table 2： References for cell type annotation. Cell marker genes and relevant references were listed.

Supplementary table 3： Differentially expressed genes in each cell subsets of mesenchyme and ectoderm cells.

Supplementary table 4： Annotation of the re-clustered mesenchyme and ectoderm showing marker genes used for mapping and assignment, respectively.

Supplementary table 5： Regulon activities for subsets of mesenchyme and ectoderm cells. TF regulon activity scores quantified by AUCell were shown. The g within the bracket indicates the number of genes in the regulons.

Supplementary table 6： Motifs of pivotal genes involved in the formation of upper lip and primary palate.
